# A preliminary investigation into bacterial viability using scanning electron microscopy–energy-dispersive X-ray analysis: The case of antibiotics

**DOI:** 10.3389/fmicb.2022.967904

**Published:** 2022-08-08

**Authors:** Gabriel Haddad, Tatsuki Takakura, Sara Bellali, Anthony Fontanini, Yusuke Ominami, Jacques Bou Khalil, Didier Raoult

**Affiliations:** ^1^Institut Hospitalo-Universitaire Méditerranée Infection, Marseille, France; ^2^Aix-Marseille Univ, IRD, APHM, MEPHI, Marseille, France; ^3^Hitachi High-Tech Corporation, Hitachinaka-shi, Japan

**Keywords:** metabolic state, chemical composition, EDX, SEM, bacterial viability, scanning electron microscopy, energy dispersive X-ray

## Abstract

The metabolic stages of bacterial development and viability under different stress conditions induced by disinfection, chemical treatments, temperature, or atmospheric changes have been thoroughly investigated. Here, we aim to evaluate early metabolic modifications in bacteria following induced stress, resulting in alterations to bacterial metabolism. A protocol was optimized for bacterial preparation using energy-dispersive X-ray (EDX) microanalysis coupled with scanning electron microscopy (SEM), followed by optimizing EDX data acquisition and analysis. We investigated different preparation methods aiming to detect modifications in the bacterial chemical composition at different states. We first investigated Escherichia coli, acquiring data from fresh bacteria, after heat shock, and after contact with 70% ethanol, in order to prove the feasibility of this new strategy. We then applied the new method to different bacterial species following 1 h of incubation with increasing doses of antibiotics used as a stress-inducing agent. Among the different materials tested aiming to avoiding interaction with bacterial metabolites, phosphorous-doped silicon wafers were selected for the slide preparation. The 15 kV acceleration voltage ensured all the chemical elements of interest were excited. A thick layer of bacterial culture was deposited on the silicon wafer providing information from multiple cells and intra-cellular composition. The EDX spectra of fresh, heat-killed, and alcohol-killed *E. coli* revealed important modifications in magnesium, potassium, and sodium. Those same alterations were detected when applying this strategy to bacteria exposed to antibiotics. Tests based on SEM–EDX acquisition systems would provide early predictions of the bacterial viability state in different conditions, yielding earlier results than culture.

## Introduction

Assessing bacterial viability is of great interest to clinical and fundamental microbiology (Caron et al., 1998; Kumar and Ghosh, 2019). Monitoring the bacterial metabolic state under different conditions has been intensely investigated. Numerous studies have explored and reported metabolic modifications in bacteria aiming to investigate and improve growth in different culture conditions, biofilm formation, and their response to chemical agents (Diaper et al., 1992; Maukonen et al., 2000; Keer and Birch, 2003; Rogers et al., 2010). Accessing this kind of information would provide better insights on the development, survival, and metabolism of bacteria, leading to a better understanding of the bacterial state and viability (Barer and Harwood, 1999). Several methods have been developed which aim to evaluate bacterial metabolic state for multiple uses in clinical and environmental microbiology, assessing the performance of disinfection shock treatment, and the detection of bacterial pathogens in food and water. Some of these methods have been based on quantifying colony formation and bacterial growth using digital microscopy and image analysis algorithms (Fredborg et al., 2013; Price et al., 2014; Le Page et al., 2015). Other approaches have used single-cell detection, combining optics and microfluidic devices to detect quantifiable (Mohan et al., 2013; Etayash et al., 2016) or morphological changes (Nilsson et al., 1991; Choi et al., 2014; Quach et al., 2016; Baltekin et al., 2017; Zahir et al., 2019). Some studies have evaluated the presence of viable microorganisms in water using direct culture or co-culture strategies (Adams et al., 2003; Delgado-Viscogliosi et al., 2005), or by amplifying and quantifying RNA (van der Vliet et al., 1994; Prudent et al., 2017). Fluorescence detection by flow cytometry or imaging has also been extensively researched and widely used for the detection and assessment of viable potential foodborne pathogens in various ecosystems (Diaper and Edwards, 1994; Boulos et al., 1999; Adams et al., 2003; Delgado-Viscogliosi et al., 2005; Brauge et al., 2019). Recently, scanning electron microscopy (SEM) has been re-evaluated for applications in clinical microbiology (Cushnie et al., 2016; Hannachi et al., 2020; Haddad et al., 2021a,b). In addition to high-resolution images, SEM can provide information on the chemical elements present in a specimen when coupled with energy-dispersive X-ray spectroscopy (EDX). An SEM–EDX system enables semi-quantitative elemental microanalysis by measuring the generation of characteristic X-rays from each chemical element present in the specimen. One recent study revealed the potential for bacterial identification by combining SEM morphological information and EDX data (Khan et al., 2020).

In this study, we investigated bacterial chemical composition and modifications to it, based on EDX coupled with a tabletop SEM. We aimed to monitor the bacterial metabolic state and detect early onset metabolic modifications of various bacterial species under stress induced by heat, disinfection, and antimicrobial agents.

## Materials and methods

### Bacterial strains collection and growth conditions

Six bacterial isolates of *Escherichia coli*, *Klebsiella pneumoniae*, and *Enterobacter cloacae* were collected from the “Collection de Souches de l’Unité des Rickettsies” (CSUR, WDCM 875; Table 1). Bacteria were identified using matrix-assisted laser desorption/ionization time-of-flight mass spectrometry (MALDI-TOF MS) on a Microflex LT spectrometer (Bruker Daltonics, United States; Seng et al., 2009). The bacterial strains were grown overnight in tryptone soya broth (TSB; Becton Dickinson, United States) at 37°C under aerobic conditions. Then, the fresh cultures were diluted using TSB and adjusted at an optical absorbance of 0.18 (10^6^–10^7^ CFU/ml), at a wavelength of 600 nm measured by an Ultrospec 10 cell density meter (Biochrom, United Kingdom). Experiments were carried out on 4 ml of bacterial suspension per condition and bacterial concentrations were validated by the colony counting method.

**Table 1:** List of bacteria and antibiotic susceptibility profiles.

Species	Strain	Susceptibility	Antibiotic	MIC	ECOFF
*Escherichia coli*	Q5586	Susceptible	Imipenem	0.25 mg/L	0.5 mg/L
	*Escherichia coli*				
P1872		Resistant	>32 mg/L
*Klebsiella pneumoniae*	Q5580	Susceptible	Imipenem	1 mg/L	2 mg/L
	*Klebsiella pneumoniae*				
Q2447		Resistant	4 mg/L
*Enterobacter cloacae*	P9549	Susceptible	Imipenem	0.25 mg/L	1 mg/L
	*Enterobacter cloacae*				
P9548		Resistant	2 mg/L

### Proof of concept

#### Conditions tested

Fresh *E. coli* bacterial suspensions were analyzed to identify their elemental EDX spectra. The same bacterial suspensions were also heat shocked at 90°C for 30 min or exposed to 70%ethanol to kill the bacteria. We aimed to detect modifications in the chemical composition of fresh and dead bacteria. All experiments were performed in triplicate.

#### SEM–EDX method

##### Optimization of sample preparation for EDX measurements

For the sample preparation support, different materials (including glass slides, phosphorous-doped silicon (Si) wafers, plastic and metallic surfaces) were assessed for minimum interaction with bacterial metabolites during EDX acquisition. The bacterial suspensions were centrifuged at 1,700 × *g* for 10 min (Centrifuge 5810R, Eppendorf, Germany) to separate bacterial pellets. After removing the supernatant, the pellets were then rinsed in 3 ml of distilled water (Bio-Rad Laboratories, United States), followed by a second centrifugation under the same conditions. Five to 10 μl of condensed bacterial suspensions were collected. The condensed bacterial suspension was applied to the surface of a 15 mm × 35 mm Silicon (Si) wafer (Siltronix, France) and dried under a biosafety hood. Dried spots of bacterial deposits with a typical thickness of 5–15 μm were obtained depending on the bacterial concentration after rinsing.

##### EDX measurement conditions: Selection of settings

Bacterial deposits were observed using the Tabletop SEM TM4000 Plus (Hitachi High-Tech, Japan) combined with the AZtecOne EDX system (Oxford Instruments, United Kingdom). The preparations were loaded into the TM4000 Plus with a 10 mm distance between the sample surface and the detector. Glucose was used as a sample material for the Monte Carlo simulation, since it has an elemental composition similar to bacterial cells. The acceleration voltage was set at 15 kV, with the highest beam current mode (LensMode 4) under vacuum conditions (<30 Pa). Each bacterial deposit was observed at ×300 magnification. Map-sum EDX spectra of each image area were taken using a mapping mode at 256 times image resolution, three times frame count, and 200 μs pixel dwell time. EDX spectra from Si substrate were also measured for spectral analysis.

##### EDX spectral analysis and calculation

To analyze elemental peaks originating from bacterial cells as accurately as possible, peaks from EDX spectra were first extracted by a numerical fitting based on the least square method (Van Grieken and Markowic, 2002; Bevington and Robinson, 2003). In our analysis, only the main Kα peaks of Si, carbon (C), oxygen (O), nitrogen (N), calcium (Ca), magnesium (Mg), sulfur (S), sodium (Na), phosphorous (P), chlorine (Cl), and potassium (K) were taken into consideration (Supplementary material; Supplementary Figure S1).

### Application: Antibiotic-induced bacterial stress

For a biochemically induced stress that would allow the investigation of progressive modifications in bacteria, we selected a carbapenem (imipenem). Bacterial strains were selected based on their susceptibility profiles against imipenem (Table 1). The antimicrobial susceptibility of the six isolates was assayed by the E-test technique (bioMérieux) using Mueller Hinton E agar (bioMérieux, France) and incubated at 37°C for between 16 and 18 h. Different antibiotic concentrations were added, varying from one-quarter to four times the epidemiological cut-off (ECOFF) for each of the species (The European Committee on Antimicrobial Susceptibility Testing, 2022; Breakpoint tables for interpretation of MICs and zone diameters). The cultures were incubated at 37°C for 60 min under agitation and then analyzed using the same strategy described above (Supplementary Figure S2).

### Method validation: Reproducibility and statistical analysis

Each measurement of individual bacterial deposits consisted of three different fields of view. The peak height of each element was calculated by averaging the three EDX spectra from each image. In-house software (MCAM version 7.0) was used for the Monte Carlo simulation to assess the electron beam scattering in the sample with different acceleration voltages (Kyser and Murata, 1974). Excel 2010/2016 was used for EDX spectral analysis and the least squares fitting to the theoretical curve. A one-way ANOVA test was applied, followed by a Tukey’s test, to compare each condition to the antibiotic-free control at *p* < 0.05 using GraphPad Prism software 5.03 (GraphPad, San Diego, CA).

## Results

### Sample preparation and EDX measurements: Selected conditions

Si was selected as a material for the substrate, given its negligible concentration in typical bacterial cells, avoiding interactions and overlapping elements between the bacteria and the slide. This overlap removal will ensure the absence of interaction between EDX signals from the substrate and the sample, thus eliminating biased results.

The acceleration voltage of the electron beam is one of the most important parameters in EDX measurement. The electron beam is scattered in the sample while generating X-rays, which are detected as an EDX signal. The Monte Carlo simulation (Supplementary Figure S3) revealed that when the acceleration voltage was 5 kV, the penetration depth of the electron beam was <500 nm, which mainly provides signal from the bacterial surface. With an acceleration voltage of 15 kV, the penetration depth was about 3 μm. With the larger penetration volume, the EDX signal is generated from more bacterial cells and thus provides averaged information from multiple cells, including the intra-cellular atomic composition. The acceleration voltage was set at 15 kV with the highest beam current mode. This setting ensured excitation of all the chemical elements of interest and obtaining as many X-ray signals as possible.

### EDX spectral analysis and calculation

The experimental EDX spectrum contained a non-bacterial contribution. First, the spectrum of the Si substrate taken from the background measurements was subtracted from all the experimental spectra to cancel out the Si peak. This background spectrum was observed as an increase in the Na, P, Cl, and K peaks, affecting the quantitative analysis of bacterial chemical components. To derive the spectrum of the bacterial deposition, the difference between the averaged spectrum of the control samples with rinsing $I_{R}\left(E\right)$ and without rinsing $I_{\mathrm{NR}}\left(E\right)$ was subtracted from the original spectrum $I_{0}\left(E\right)$ . This correction procedure enabled the direct comparison of EDX spectra under different conditions.

### Metabolic modification in *E. coli* after heat shock

**Figure 1:**
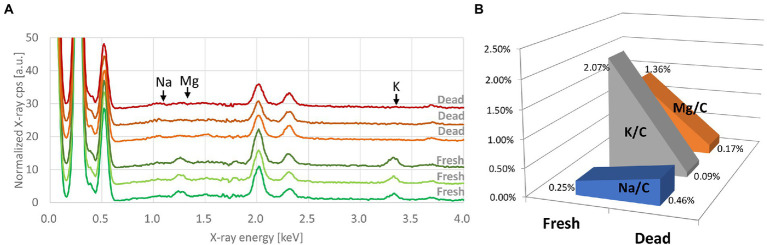
**(A)** Processed EDX spectra of fresh and heat-killed *Escherichia coli* (Q5586). Each curve represents the average of three spectra taken from the same bacterial deposition. Spectra are offset by 3. **(B)** Evolution of the K/C, Mg/C, and Na/C ratios between the fresh and the heat-killed *E. coli*.

Major chemical elements observed in the spectra were C, O, and P. Other minor elements were also detected, namely N, Ca, Mg, S, K, and Na. Processed EDX spectra revealed alterations in the bacterial elemental composition for Mg, K, and Na between the fresh and heat-killed bacteria (Figure 1A), which we correlated to the metabolic response of the bacteria to the heat shock. Judging from these observations, Mg, K, and Na concentrations were used as chemical indicators of bacterial metabolic modifications (Figure 1B).

### Metabolic modification in *E. coli* after exposure to alcohol

**Figure 2:**
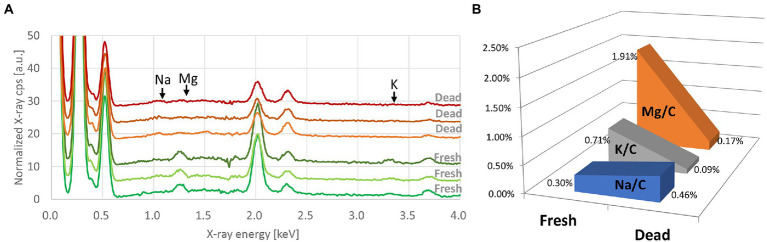
**(A)** Processed EDX spectra of *E. coli* (Q5586) at the fresh state and after exposure to 70% ethanol. Each curve represents the average of three spectra taken from the same bacterial deposition. Spectra are offset by 3. **(B)** Evolution of the K/C, Mg/C, and Na/C ratios between the fresh and the alcohol-killed *E. coli*.

C, O, and P were the most common chemical elements present on the EDX spectra. Traces of other elements were also detected. We found the same differences in bacterial elemental composition, mainly for Mg, K, and Na, between fresh bacteria and those exposed to 70% ethanol (Figure 2). Based on our results, these elements were adopted as chemical markers of bacterial metabolic changes.

### Metabolic modification in bacteria after antibiotic treatment

**Figure 3:**
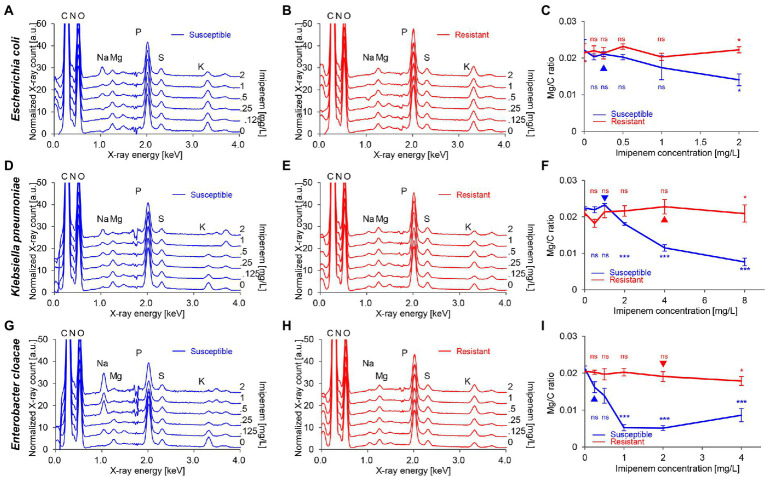
EDX spectra and Mg/C ratio of Gram-negative bacilli incubated with imipenem. EDX spectra of **(A, D, G)** susceptible (Q5586, Q5580 and P9549) and **(B, E, H)** resistant (P1872, Q2247, and P9548) strains of *E. coli*, *Klebsiella pneumoniae* and *Enterobacter cloacae*, respectively. Each curve represents the average of three spectra taken from the same bacterial deposit. Spectra are offset by 5 **(C, F, I)** Mg/C ratio of tested strains calculated from the respective EDX spectra. Error bars: standard deviations. Blue and red triangles: MIC of tested strains. ^*^p < 0.01; ^**^p < 0.001; ^**^p < 0.0001; (ns): not significant.

**Figure 4:**
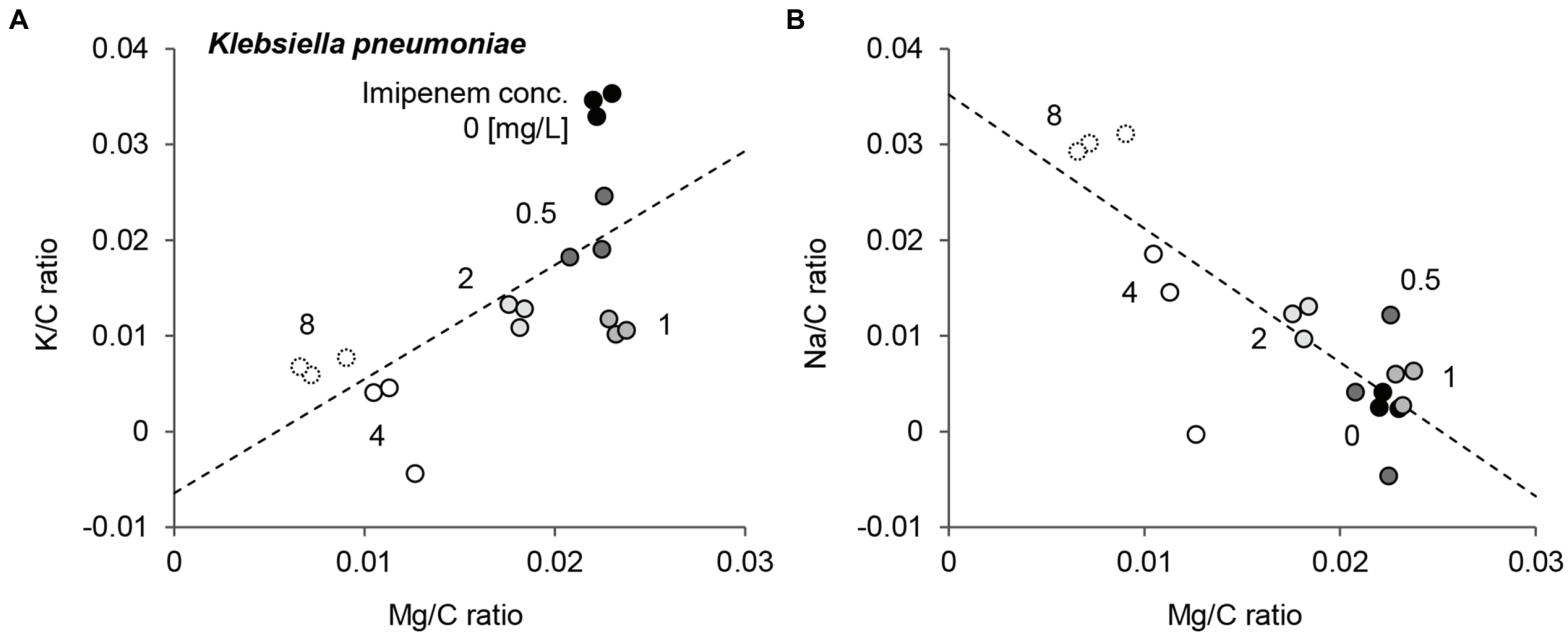
Comparison of Mg/C ratio, K/C ratio, and Na/C ratio of a *Klebsiella pneumoniae* susceptible strain incubated with various concentrations of imipenem. K/C ratio **(A)** and Na/C ratio **(B)** as a function of Mg/C ratio. Numbers in the figure represent the imipenem concentration. Dashed lines are linear fitting for all plots.

EDX spectra of *E. coli*, *K. pneumoniae*, and *E. cloacae* incubated without and with increasing imipenem concentrations were acquired (Figure 3). In some cases, aluminum (Al) peaks (1.49 keV) were detected, owing to the interaction between scattered electrons and the sample support made of Al. Signal fluctuation around 1.74 keV resulted from the subtraction of Si peaks. Obvious decreases in the Mg and K peaks, and a rise in the Na peak were also detected for the susceptible strains (*E. coli* Q5586, *K. pneumoniae* Q5580, and E. cloacae P9549), with increasing imipenem concentrations (MIC: 0.25, 1 and 0.25 mg/L respectively; Figures 3A,D,G). We correlated these modifications to the metabolic response of the bacteria to the induced chemical stress. However, these modifications were not observed in the resistant strains (*E. coli* P1872, *K. pneumoniae* Q2247, and E. cloacae P9548; MIC: >32, 4 and 2 mg/L respectively; Figures 3B,E,H).These results were confirmed by culture and SEM imaging. However, the K and Na concentrations express a high variability among the spectra from the same bacterial deposit, due to their presence in the culture medium. Therefore, the measurement of Mg was selected as the follow-up chemical element for our metabolic profiling. Regarding the susceptible strains Q5586, Q5580, and P9549, the Mg/C ratio showed no significant change from the control (*p* > 0.05) below the MIC. Exceeding this limit, the Mg/C ratio tends to decrease monotonically with increasing imipenem concentrations, which was more pronounced in the case of K. pneumoniae and E. cloacae (*p* < 0.0001; Figures 3C,F,I). The Mg/C ratio of the resistant strains showed no significant changes compared to the imipenem-free controls below the MIC, where K. pneumoniae Q2247 and E. cloacae P9548 showed a decreasing tendency which is less prominent than the susceptible isolates (*p* < 0.01 and *p* > 0.05, respectively; Figures 3C,F,I). These results indicate that the antibiotic-concentration dependency of the Mg/C ratio corresponds well to conventional MICs. When evaluating the correlation of the K/C and Na/C ratios with Mg/C for *K. pneumoniae* Q5580, the plots were distributed along a line with a positive and negative slope, respectively (Figure 4). These correlations imply the leakage of cytoplasmic cations caused by bacterial lysis. Similar correlations were also observed in the case of *E. coli* Q5586 and *E. cloacae* P9549 (not shown).

## Discussion

In our study, we optimized a method for the rapid preparation of bacterial cultures on Si wafers, followed by EDX spectral acquisition and analyses to evaluate early metabolic modifications. Monitoring and understanding bacterial metabolic alterations imply comparing bacteria under stress conditions induced by temperature change, disinfection, and biochemical treatments. Our choice of antibiotics to study the metabolic changes in bacteria reveals details with progressive changes at increasing concentrations (Hanberger et al., 1991; Cushnie et al., 2016; Haddad et al., 2021b). When the cell wall is damaged, pores are created, leading to diffusive ion movement, resulting in a decrease of K^+^ and Mg^2+^ and an increase in Na^+^(Lu et al., 2017). The integrity of the bacterial cell wall relies on lipid ordering and bilayer stability, both affected by heat or solvents (de Kruijff et al., 1985; Yura et al., 1993; Ramos et al., 2001). Also, increasing Mg^2+^ was correlated with the dividing phase of E. coli in actively dividing cultures (Chang et al., 1986). In this study, we confirmed these results by detecting a dissipation of the Mg^2+^ peak after bacterial death. Moreover, Chang et al. reported the reduction in monovalent ions signals after washing EDX substrates with distilled water, while cellular Mg and membranous Ca seemed more tightly bound and conserved (Chang et al., 1986). Therefore, leakage of cytoplasm caused by cell lysis in stressful conditions is the main cause of the decrease in Mg, which also corresponds to the cell lysis of the isolates when incubated with antibiotics (Hanberger et al., 1991; Le Page et al., 2015). EDX spectra complement the SEM investigations, especially in the case of delayed or non-visible morphological alterations, depending on the micro-organism tested. However, our method remains limited, since the sample preparation was optimized on a thick layer of bacteria from pure cultures. Further investigations are needed to apply this method directly to a given sample, accounting for interference of the EDX spectrum from the culture medium and other components. One way to resolve this issue would be a complete separation of bacterial cells from the remaining culture medium and other components, for an accurate analysis of the signal. On the other hand, this method proved to be efficient at investigating and detecting early onset metabolic modifications in bacterial composition induced by heat, disinfection, and antimicrobial agents, giving robust and consistent results, thus providing predictive information on bacterial metabolic state and viability, and yielding earlier results than culture. In this work, EDX coupled to SEM proved its ability capability of detecting non-morphological antibiotic effects at early stages of the bacterial growth when incubated with antibiotics, opening a new strategic path in assessing the early bacterial response. This assay presents a potential candidate for the development rapid antibiotic susceptibility testing applicable in clinical microbiology. Furthermore, automatically identifying the field of view will be easily achievable by depositing the bacterial suspension at reproducible positions with an optimal design of the substrate. Shortening the preparation and EDX acquisition time, as well as system automation, should be considered as key factors for future potential implementation of our newly developed strategy.

## Data availability statement

The original contributions presented in the study are included in the article/Supplementary material, further inquiries can be directed to the corresponding authors.

## Author contributions

GH, TT, SB, and AF performed the experiments. GH, TT, and SB analyzed the data and wrote the paper. SB, JK, YO, and DR revised the paper. DR and JK supervised the study. All authors contributed to the article and approved the submitted version.

## Funding

This work was supported by a grant from the French Government managed by the National Research Agency under the “Investissements d’avenir” (Investments for the Future) programme with the reference ANR-10-IAHU-03 (Méditerranée Infection), by the Région Provence-Alpes-Côte-d’Azur and European ERDF PRIMI funding. In addition, collaborative study between IHU Méditerranée Infection and the Hitachi High-Tech Corporation is funded by the Hitachi High-Tech Corporation.

## Conflict of interest

The authors would like to declare that DR was a consultant in microbiology for the Hitachi High-Tech Corporation between March 2018 and March 2021. TT and YO were employed by the Hitachi High-Tech Corporation.

The remaining authors declare that funding sources played no role in the design and conduct of the study, the collection, management, analysis, and interpretation of the data, nor in the preparation, review, or approval of the manuscript.

## Publisher’s note

All claims expressed in this article are solely those of the authors and do not necessarily represent those of their affiliated organizations, or those of the publisher, the editors and the reviewers. Any product that may be evaluated in this article, or claim that may be made by its manufacturer, is not guaranteed or endorsed by the publisher.
