# Supplementary material for: A preliminary investigation into bacterial viability using scanning electron microscopy–energy-dispersive X-ray analysis: The case of antibiotics
Source: Front Microbiol. 2022 Aug 8;13:967904. doi: 10.3389/fmicb.2022.967904 (PMC9393632; doi:10.3389/fmicb.2022.967904)
Supplement: Supplementary file 1 [file Data_Sheet_1.docx]

**Supplementary figures**

**Figure S1. Overview of EDX spectral analysis.** **(Step 1).** Each peak height is extracted by the numerical fitting with the equation (e.1). **(Step 2).** The background spectra from the substrate and the culture medium are subtracted from the original spectrum of the target sample by cancelling out the *Si* and *Cl* peaks. **(Inset)** Black curve: example of EDX spectra before background correction. Blue curve: example of EDX spectra after background correction.

**Figure S2. Overview of sample preparation.** **(a)** Flow chart of sample preparation for antibiotic analysis. The bacterial separation and rinsing step include two centrifugation cycles. **(b)** Photograph of silicon substrate with bacterial deposition. Dashed yellow circles: bacterial deposits with six different antibiotic concentrations. A control sample without rinsing is used for background correction.

**Figure S3.** Two-dimensional Monte Carlo simulation of the electron beam scattering in the sample with 5 kV **(a)**, 10 kV **(b)**, and 15 kV **(c)** of acceleration voltage. Glucose as a sample material was assumed to imitate bacterial cells. White areas: vacuum; blue areas: glucose. Red lines: trajectory of incident electron beam. blue curves: trajectories of scattered electrons. Scale bars: 1 μm.

**Supplementary material**

***EDX spectral analysis and calculation***

The following theoretical model was used to describe an experimental EDX spectrum:

$I_{\mathrm{EDX}}\left( E \right)=K\left( E_{\max}-E \right)^{\alpha}e^{-\frac{\beta}{E^{3}}}+\sum_{i} c_{i}e^{-\frac{\left( E-E_{i} \right)^{2}}{\sigma^{2}}}$,

where $I_{\mathrm{EDX}}\left( E \right)$ is the EDX spectrum, $E$ is the energy of the X-ray, $E_{\max}$ is the maximum energy of the X-ray (15 keV), and $E_{i}$ is the energy of a characteristic X-ray from i-th chemical element, respectively. Here$K$, $\alpha$, $\beta$, $c_{i}$, and $\sigma$ are fitting parameters, and$c_{i}$ corresponds to the peak size of i-th chemical element, which is proportional to the concentration of the element. The first term in the equation is a modified classical Kramers model considering energy-dependent X-ray absorption in the sample. The second term represents the sum of characteristic X-rays from each chemical element with a Gaussian energy distribution.

***Calculations to derive the spectrum of the bacterial deposition***

$I_{\mathrm{bacteria}}\left( E \right)=I_{0}\left( E \right)-\boldsymbol{c}\left( I_{\mathrm{NR}}\left( E \right)-I_{R}\left( E \right) \right)$.

Here $\boldsymbol{c}$ is the constant that is determined to cancel out the *Cl* peak of $I_{0}\left( E \right),$ as the *Cl* concentration is expected to be much lower than that of other elements in rinsed bacteria (Chang et al., 1986b). After these subtractions, elemental peak sizes were normalized by that of *C*, because the intensity of the spectrum was proportional to the density of the bacterial deposit.
