# Supplementary figures and images for: A preliminary investigation into bacterial viability using scanning electron microscopy–energy-dispersive X-ray analysis: The case of antibiotics
Source: Front Microbiol. 2022 Aug 8;13:967904. doi: 10.3389/fmicb.2022.967904 (PMC9393632; doi:10.3389/fmicb.2022.967904)

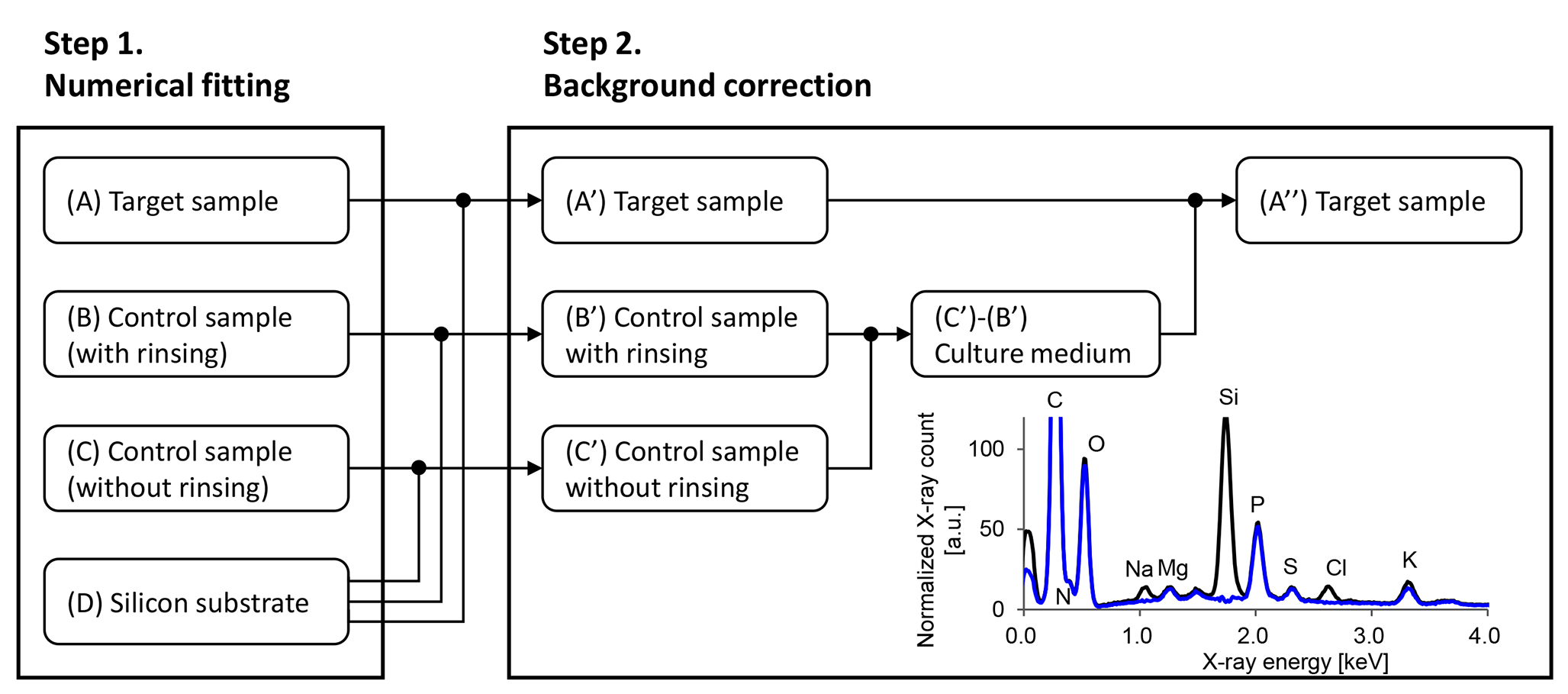

Supplement: Supplementary file 2 [file Image_1.TIF]

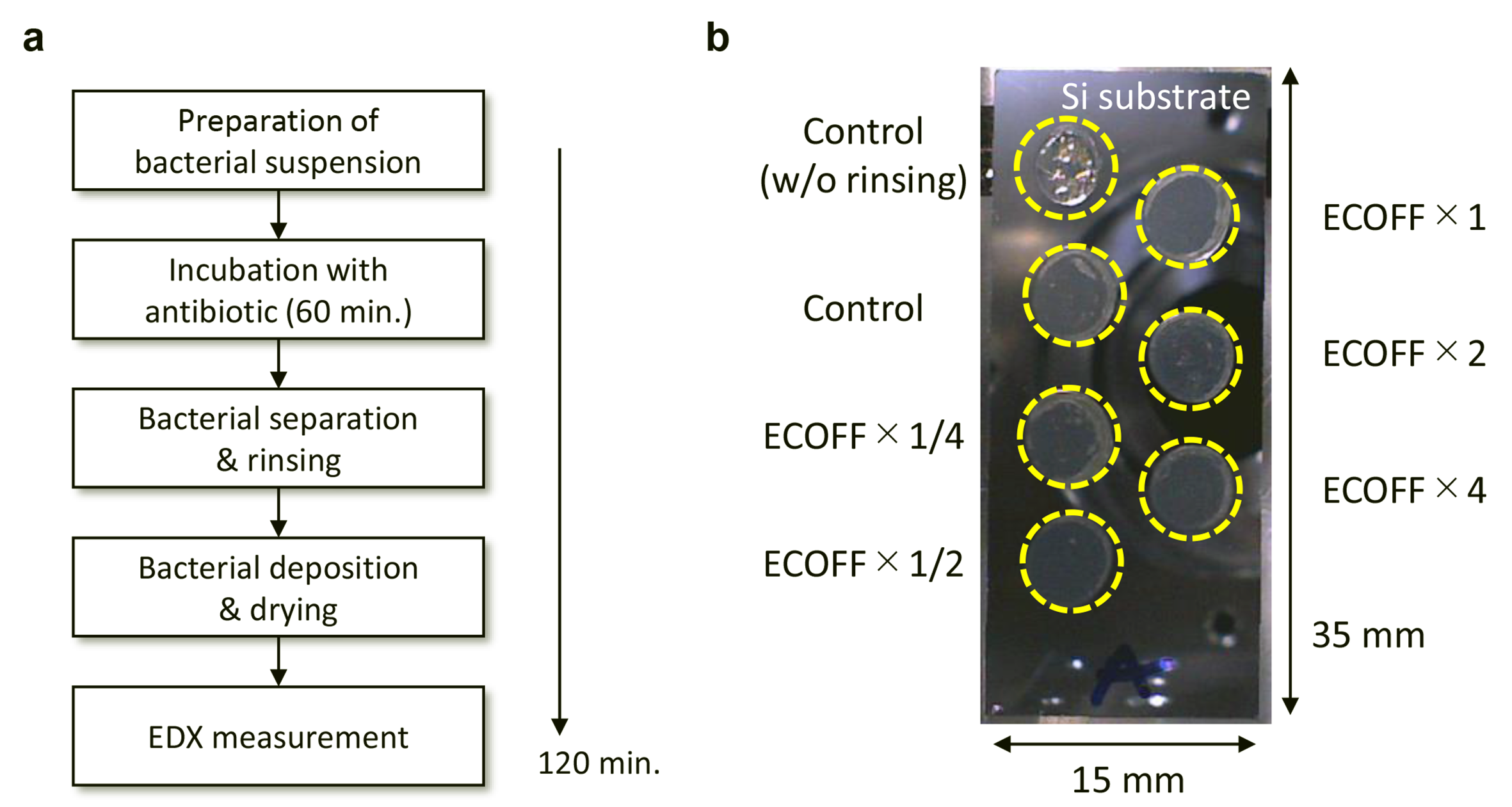

Supplement: Supplementary file 3 [file Image_2.TIF]

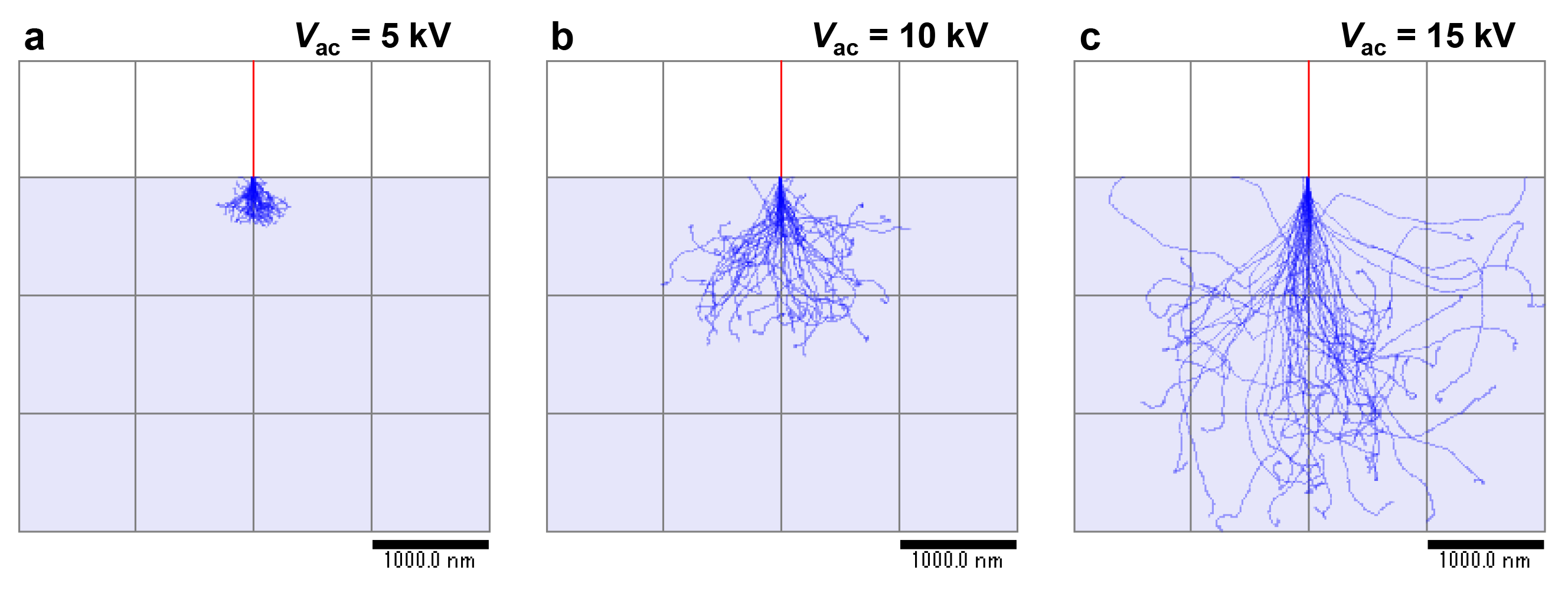

Supplement: Supplementary file 4 [file Image_3.TIF]
